# Next-Generation Sequencing (NGS) Utility in Advanced Solid Tumors in Low- and Middle-Income Country (LMIC) Setting

**DOI:** 10.7759/cureus.113197

**Published:** 2026-07-22

**Authors:** Prakriti Singh, Abhinav Manish, Amit Badola, Tanvi Khanna

**Affiliations:** 1 Medical Oncology, Asian Institute of Medical Sciences, Faridabad, IND; 2 Biochemistry, Gautam Buddha Chikitsa Mahavidyalaya, Dehradun, IND; 3 Medical Oncology, Max Super Speciality Hospital, Dehradun, IND; 4 Paediatrics, Shri Guru Ram Rai (SGRR) Institute of Medical and Health Sciences, Dehradun, IND

**Keywords:** genomic profiling, lmic, next-generation sequencing, precision oncology, real-world data, resource-limited setting, solid tumors

## Abstract

Introduction

Advancements in DNA sequencing have revolutionized our understanding of cancer biology, enabling comprehensive analysis of genetic alterations driving tumorigenesis. Next-generation sequencing (NGS) is increasingly integrated into oncology practice to identify actionable mutations and personalize therapy. However, its clinical utility in low- and middle-income countries (LMICs), where access to sequencing technologies and targeted treatments remains limited, is not well established.

Methods

This was a retrospective, single-center cohort study involving adult patients with advanced metastatic solid tumors who underwent NGS testing using either tumor tissue or liquid biopsy (Illumina platform, 560-gene panel, 1000X coverage). The primary endpoint was to evaluate the proportion of patients who received NGS-guided targeted therapy. The secondary endpoint was progression-free survival (PFS) in patients treated based on NGS findings. Data analysis was performed using Microsoft Excel 2016 (Microsoft Corp., Redmond, WA, USA).

Results

Over an 18-month period (January 2021 to June 2022), 93 patients were included. Genomic alterations were detected in 45% (n=42) of patients. Among these, 33% (n=31) had a potentially actionable mutation with an available targeted therapy or clinical trial option. Despite this, only 19% (n=18) received NGS-directed treatment. Of these, 44% (n=8) achieved a PFS of six months or more.

Conclusion

NGS can identify clinically relevant mutations in a significant proportion of advanced cancer patients in LMIC settings. However, real-world application is hindered by limited access to targeted therapies and clinical trials. Strategies to improve access and infrastructure are essential to realize the full potential of precision oncology in these regions.

## Introduction

Cancer remains one of the leading causes of morbidity and mortality globally. Despite notable advances in systemic therapy, the prognosis for patients with advanced metastatic solid tumors continues to be poor, primarily due to the inability of conventional treatments - surgery, radiotherapy, and chemotherapy - to effectively target the molecular drivers of malignancy.

Recent advancements in cancer genomics have revolutionized oncology by enabling more precise and individualized therapeutic strategies. Among these innovations, next-generation sequencing (NGS) has emerged as a powerful tool for comprehensive molecular profiling. Unlike first-generation sequencing technologies, NGS allows for rapid, high-throughput analysis of entire genomes, exomes, or targeted gene panels, using either tumor tissue or circulating tumor DNA from blood samples [1].

The widespread adoption of NGS has provided invaluable insights into tumor biology and has enabled the identification of prognostic, diagnostic, and predictive biomarkers. Multiple studies have demonstrated the utility of NGS in detecting actionable driver mutations in various cancers, including lung, breast, and colorectal malignancies, leading to the successful implementation of targeted therapies that have improved patient outcomes [2,3].

In recognition of the growing relevance of molecular oncology, the European Society for Medical Oncology (ESMO) developed the ESMO Scale for Clinical Actionability of Molecular Targets (ESCAT), a systematic framework for classifying genomic alterations based on the strength of clinical evidence supporting targeted treatment [4]. While such frameworks support evidence-based application of NGS in clinical practice, their real-world implementation - especially in resource-constrained settings - remains variable and sometimes uncertain [5].

In high-income countries, NGS is increasingly integrated into routine oncology care, guiding treatment decisions for patients with advanced or refractory cancers. However, in low- and middle-income countries (LMICs), the clinical utility of NGS is still evolving. Challenges such as limited access to sequencing platforms, high costs, lack of reimbursement, and restricted availability of matched targeted therapies continue to impede its broader adoption [6].

In this study, we present real-world data from a single-center experience in an LMIC, evaluating the impact of NGS-guided treatment on clinical outcomes - specifically, progression-free survival (PFS) - in patients with advanced solid tumors.

## Materials and methods

This was a single-centre, retrospective cohort study conducted over one year, from January 1, 2021, to June 30, 2022. A total of 93 patients with advanced metastatic solid tumours were included in the study. The study included adult patients with advanced or metastatic solid tumours who underwent next-generation sequencing (NGS) either on archived tumour tissue blocks or via liquid biopsy.

Patients known to harbour genetic alterations for which standardised, guideline-recommended targeted therapies already exist were excluded. This included, but was not limited to, EGFR exon 18, 19, and 21 mutations in non-small cell lung cancer; HER2 amplification and PIK3CA mutations in breast cancer; BRAF mutations in melanoma and lung cancer; KRAS mutations in colorectal cancer; and KIT mutations in gastrointestinal stromal tumours.

NGS was performed using a commercially available 560-gene panel (Illumina platform) with 1000X coverage. The assay was capable of detecting various types of genomic alterations, including single-nucleotide variants, insertions, deletions, fusions, amplifications, rearrangements, and frameshift mutations. The generated reports identified actionable mutations and suggested potential targeted therapies, including both approved drugs and those available through clinical trials.

The primary endpoint was to assess the clinical utility of NGS by determining the proportion of patients who received genome-directed therapy based on actionable mutations identified. The secondary endpoint was to evaluate clinical benefit, as measured by progression-free survival (PFS) in patients receiving NGS-guided therapy. All statistical analyses were performed using Microsoft Excel 2016.

## Results

Among 93 patients with advanced metastatic solid tumours who were included in the study, their baseline characteristics are summarised in Table 1. Out of enrolled patients, 60% (n=56) were male, and 40% (n=37) were female. Lung cancer was the most common malignancy, observed in 23% (n=21) of patients, followed by gallbladder cancer in 12% (n=11), and both breast and head and neck cancers, each accounting for 11% (n=10).

NGS testing was performed at various stages of treatment: before first-line therapy in 32% (n=30), second-line in 46% (n=43), and third-line or later in 22% (n=20) of patients (Table 1).

**Table 1:** Baseline characteristics of study population (n=93) NGS: Next-generation sequencing; PFS: Progression-free survival.

Baseline characteristics				Percentage (number of patients)
Gender			Male	60% (n=56)
			Female	40% (n=37)
Type of Biopsy	Tissue			71% (n=66)
	Liquid			29% (n=27)
Genetic alteration present				45% (n=42)
Timing of NGS testing		1^st^ line		32% (n=30)
		2^nd^ line		46% (n=43)
		3^rd^ line or beyond		22% (n=20)
Targetable approved treatment or clinical trial available				33% (n=31)
Patient received treatment as per molecular alteration				19% (n=18)
PFS >6 months in molecular alteration-guided treatment				9% (n=8)

Genomic alterations were identified in 45% (n=42) of patients. Among these, single-nucleotide variants (SNVs) were the most common, detected in 65% of cases. Gene amplification and deletion were observed in 13% and 12% of patients, respectively, while 10% had frameshift mutations.

Of the 42 patients with detectable genomic alterations, 33% (n=31) had potentially actionable mutations with a corresponding targeted therapy available, either as an approved treatment or through a clinical trial. Despite this, only 19% (n=18) of patients ultimately received NGS-directed therapy, as detailed in Table 2.

**Table 2:** Details of patients who received NGS-directed treatment NGS: Next-generation sequencing; PFS: Progression-free survival.

S.N.	Cancer Type	Lines of systemic therapy before NGS testing	Genetic alteration detected	NGS-directed treatment	PFS
1	Ca Lung	2	TMB-H	Keytruda	<6 months
2	Ca Lung	2	EGFR amplification	Everolimus	<6 months
3	Ca Lung	2	TMB-H	Keytruda	>6 months
4	Ca Lung	2	PIK3R1	Everolimus	<6 months
5	Ca Gallbladder	1	TMB-H	Keytruda	>6 months
6	Ca Gallbladder	2	Her2Neu amplification	Trastuzumab	>6 months
7	Ca Gallbladder	0	TMB-H	Keytruda	>6 months
8	Ca Gallbladder	1	TMB-H	Keytruda	<6 months
9	Ca Gallbladder	1	Microsatellite unstable	Keytruda	>6 months
10	Ca Colon	2	Her2Neu amplification	Trastuzumab and lapatinib	<6 months
11	Ca Endometrium	2	Her2Neu amplification	Trastuzumab	<6 months
12	Ca Endometrium	1	Microsatellite unstable	Keytruda	>6 months
13	Ca Esophagus	2	EGFR amplification	Gefitinib	<6 months
14	Ca Esophagus	2	SDHA mutation	Temozolomide	<6 months
15	Ca Pancreas	1	Microsatellite unstable	Keytruda	<6 months
16	Glioma	1	TMB-H	Keytruda	>6 months
17	Ca stomach	2	TMB-H	Keytruda	>6 months
18	Synovial Sarcoma	2	NF-1	Trametinib	<6 months

Among those who received targeted treatment, 44% (n=8) experienced a progression-free survival (PFS) of six months or more, while the remaining 56% (n=10) progressed within six months. Thirteen patients with actionable mutations could not be treated with genome-matched therapy - nine due to the unavailability of relevant clinical trials in India, and four because of financial constraints.

## Discussion

This study provides real-world evidence regarding the clinical utility of next-generation sequencing (NGS) in patients with advanced metastatic solid tumors in a low- and middle-income country (LMIC) setting. Our findings demonstrate that although 45% of patients had detectable genomic alterations, only 19% received NGS-directed treatment, and merely 9% achieved a progression-free survival (PFS) of six months or more.

The rate of actionable alterations (33%) in our study is consistent with previously published data. For example, the MOSCATO-01 trial reported actionable alterations in approximately 39% of advanced cancer patients, with 24% receiving matched targeted therapy and a 7% improvement in progression-free survival compared to prior therapy [7]. Similarly, the ProfiLER study showed 30% of patients had a targetable alteration, but treatment modification occurred in only 6% of cases, primarily due to logistical and economic limitations [8].

The discrepancy between the detection of actionable mutations and the actual delivery of genome-directed therapy remains a significant concern, especially in LMICs. In our cohort, 13 patients were unable to receive targeted treatment despite the availability of molecular options, owing to the unavailability of clinical trials in India and financial constraints. This highlights a persistent issue in precision oncology: access to therapy often lags behind diagnostic advances [9].

Furthermore, patients receiving NGS-matched therapy before the second-line setting seemed to have better outcomes, as observed in several cases with microsatellite instability (MSI-H) and high tumor mutational burden (TMB-H) responding to immune checkpoint inhibitors. These biomarkers are known to predict benefit from agents like pembrolizumab, which is FDA-approved for MSI-H and TMB-H tumors regardless of tissue origin [10,11]. However, broad implementation of such biomarkers in LMICs is challenged by cost, infrastructure, and drug availability [12].

The relatively low impact of NGS on therapeutic decision-making in our study is comparable to other real-world analyses. A study by Mateo et al. concluded that only 11% of patients in their cohort received matched therapy based on NGS results [4]. The ESCAT (ESMO Scale for Clinical Actionability of Molecular Targets) also emphasizes the need to prioritize alterations with high levels of evidence for clinical actionability [2]. Nonetheless, we excluded common targetable mutations with established standard-of-care therapies (e.g., EGFR, HER2, BRAF), which could explain our relatively lower PFS benefit rate.

An additional observation is that patients with gallbladder cancer constituted a notable proportion of those with actionable alterations and favorable responses. Although gallbladder cancer is relatively underrepresented in global genomic databases, emerging evidence suggests a role for HER2 amplification and MSI-H status as potential targets in this disease [13].

Overall, while the use of NGS in routine oncology practice in India is increasing, its full potential is yet to be realized. Financial barriers, lack of clinical trial access, and logistical issues in drug procurement continue to limit the real-world translation of genomic insights into clinical benefit [6].

Our study highlights the modest yet meaningful impact of NGS in guiding treatment for patients with advanced solid tumors in a resource-constrained setting. While genomic alterations were detected in nearly half of the patients, only a minority could access targeted therapies due to limited drug availability and cost issues. Nonetheless, a subset of patients receiving genome-driven therapy achieved durable responses.

To bridge the gap between molecular diagnostics and precision therapy in LMICs, there is a need for broader access to affordable targeted drugs, enrollment in international clinical trials, and policy frameworks that support precision oncology. Future studies should aim to integrate NGS with multidisciplinary decision-making and cost-effectiveness analyses to better define its utility in real-world oncology practice.

Limitation of the study

Non-availability of NGS at many superspecialty centers is the major setback right now in developing countries.

## Conclusions

NGS can identify clinically relevant mutations in a significant proportion of advanced cancer patients in LMIC settings. However, real-world application is hindered by limited access to targeted therapies and clinical trials. Strategies to improve access and infrastructure are essential to realise the full potential of precision oncology in these regions.
